# Bedeutung digitaler Medien in der Kinder- und Jugendarbeit: Digitalisierung und soziale Teilhabe als Herausforderung für Pädagog_innen

**DOI:** 10.1007/s12054-022-00534-8

**Published:** 2023-01-03

**Authors:** Maria Neumann

**Affiliations:** Martin-Luther-Universität Halle-Wittenberg, Halle, Deutschland

**Keywords:** Digitalisierung, Soziale Ungleichheit, Jugend, Covid-19-Pandemie, Professionelles Selbstverständnis, Kinder- und Jugendarbeit, Ländlicher Raum

## Abstract

Der Beitrag thematisiert die Bedingungen und Praktiken von Digitalisierungsbemühungen von Fachkräften in der Kinder- und Jugendarbeit. Der Schwerpunkt liegt hierbei auf der Bedeutung von Digitalisierungsprozessen für die gesellschaftliche Teilhabe von Jugendlichen und die Reproduktion sozialer Ungleichheiten. Hierfür werden Expert_innen-Interviews, die sich in der kulturellen Kinder- und Jugendarbeit sowie im ländlichen Raum verorten, herangezogen.

Die Covid-19-Pandemie verdeutlicht, wie stark die gesellschaftlichen Handlungspraktiken bereits von digitalen Technologien durchdrungen sind. Allerdings wurde auch sichtbar, dass diese in professionellen pädagogischen Handlungsräumen noch eher selten das Geschehen bestimmen. Im Beitrag werden die digitalen Entwicklungen in pädagogischen Einrichtungen – die während der Pandemie angestoßen wurden – exemplarisch anhand von empirischem Material in den Blick genommen und vor dem Hintergrund der Diskussion um Digitalisierung sowie sozialer Ungleichheit und professionellen Selbstverständnissen beleuchtet.

Kinder und Jugendliche wachsen heutzutage in mediatisierten Lebenswelten auf. Das bedeutet, dass ihr Alltag von Medien durchdrungen ist und diese selbstverständlich dazugehören. Insbesondere digitale Medien bestimmen verschiedene Kommunikationsformen, sowohl interpersonal als auch interaktiv. So werden Medien nicht nur zur Information oder Unterhaltung rezipiert, sondern auch zum persönlichen Austausch, gemeinsamen Spielen oder auch zu Positionierungen in öffentlichen Debatten genutzt. „Darüber werden neue Raumerfahrungen und -bezüge, Spielorte und -settings sowie soziale Begegnungen möglich.“ (vgl. Tillmann und Hugger 2014, S. 31). Mit zunehmendem Alter bewegen sich Jugendliche autonomer in den digitalen Medien außerhalb der Kontrolle der Eltern (vgl. BMFSFJ 2020, S. 57). Eine wichtige Rolle spielen dabei die sogenannten Sozialen Medien. Jugendliche nutzen diese vor allem, um am Alltag anderer teilzuhaben, dem Wunsch nach Gemeinschaft und Verbundenheit nachzugehen, nach Anerkennung zu suchen und ihre Beziehungen zu pflegen. Digitale Medien sind damit auch Medien, die zunehmend soziale Teilhabe gewährleisten (vgl. Tillmann 2020, S. 91f.).

Aber nicht nur die soziale, sondern auch die Bildungsteilhabe und damit grundlegend gesellschaftliche sowie kulturelle Teilhabe sind zunehmend mehr an digitale Medien gebunden. Digitale Kanäle werden zu zentralen Informations- und Lernmedien sowie zu Teilhabemedien an politischen und gesellschaftlichen Diskursen (vgl. BMFSFJ 2020, S. 57). Im Hinblick darauf wird in „der Sozialen Arbeit […] die These diskutiert, dass digitale Angebotsformen ein lebensweltnahes und damit prinzipiell ungleichheitsüberwindendes Potenzial haben“ (Iske & Kutscher 2020, S. 123). Überträgt man den individuellen und emanzipativen Anspruch des Ansatzes der Lebensweltorientierung nach Hans Thiersch (2014) auf die digitale Lebenswelt von Jugendlichen, gilt es, in der Sozialen Arbeit subjektorientiert danach zu fragen, wie Individuen die digitalen Medien in ihrem Alltag nutzen und welche Rolle diese in der Beziehungsgestaltung sowie Problembewältigung spielen. Der lebensweltorientierte Ansatz erfordert auch, die individuellen Teilhabemöglichkeiten und sozialen Ungleichheitsstrukturen in den Blick zu nehmen (vgl. Tillmann 2020, 96 f.; Iske & Kutscher 2020, S. 123 f.). Unter anderem wurde im 16. Kinder- und Jugendbericht festgehalten, dass die digitalen Möglichkeitsräume bzw. das Internet auch im Rahmen von pädagogischen Angeboten nicht von allen Jugendlichen gleichermaßen genutzt werden können (vgl. BMFSFJ 2020, S. 59 f.). Im Sinne des sogenannten „Digital Divide“ (Digitale Spaltung) wird sichtbar, dass Jugendliche aus höheren sozialen Milieus unter anderem über bessere Fähigkeiten in der Computerverwendung verfügen und den virtuellen Raum in einer größeren Bandbreite nutzen, bspw. für politisches Engagement, als es weniger privilegierten Jugendlichen möglich ist (BMFSFJ 2020, S. 57). Neben den unterschiedlichen Wissens- und Kompetenzrepertoires gibt es auch Differenzen bei der Qualität der technischen Ausstattung wie auch der Datenzugänge. Diese Aspekte sind von Kategorien wie Bildung, Haushaltseinkommen, Alter und Regionalität beeinflusst (vgl. Verständig et al. 2016, S. 51). Beim Regionalitätsaspekt spielt insbesondere die Netzabdeckung in den jeweiligen Wohnorten eine relevante Rolle. Im Rahmen unseres Forschungsprojekts, das in zwei als ländlich-peripher kategorisierten Regionen angesiedelt ist, werden bspw. die infrastrukturellen Ungleichheiten aufgrund des fehlenden Breitbandausbaus immer wieder thematisiert.

Für die ungleichen digitalen Teilhabechancen werden zwei Erklärungsansätze diskutiert (vgl. Iske & Kutscher 2020, S. 119). Zum einen das sogenannte „Differenzierungsparadigma“ und zum anderen das „Kohärenzparadigma“. Beim Differenzierungsparadigma werden die Ungleichheiten individualistisch begründet. Das Kohärenzparadigma beruht hingegen auf einer strukturellen Sichtweise. So werden die Teilhabemöglichkeiten von Jugendlichen in Onlineformaten im Anschluss an das Kapitalkonzept von Pierre Bourdieu (1983) abhängig von den Herkunftsressourcen, wie dem ökonomischen, sozialen und kulturellen Kapital, interpretiert.

Infolgedessen wird gefordert, dass in der Sozialen Arbeit das Phänomen der digitalen Ungleichheit stärker in den Blick genommen werden muss und danach zu fragen ist, wie pädagogische Fachkräfte bzw. Institutionen dem entgegenwirken können (vgl. Iske & Kutscher 2020, S. 123 f.), um Jugendlichen gesellschaftliche Teilhabe zu ermöglichen. Auch wenn dabei primär gefordert wird, dass Sozialpädagog_innen die Möglichkeiten ihrer Adressat_innen reflektieren, über digitale Medien an Kultur und Gesellschaft teilzuhaben, verweisen Iske und Kutscher auch auf die Verantwortung der organisationalen Ebene der Einrichtungen. Nicht allein die Fachkräfte sind hier in die Pflicht zu nehmen, sondern es bedarf gleichzeitig einer organisationalen Flankierung in Form von materieller und personeller Infrastruktur sowie der Ermöglichung von Weiterbildungen (vgl. Iske & Kutscher 2020, S. 125 f.). Die Entwicklung von digitalen Angeboten für Jugendliche setzt dabei nicht nur voraus, sich mit deren lebensweltlicher Realität auseinanderzusetzen. Es geht auch darum, digital basierte mediale Zugänge in ihrer kulturellen Relevanz und Risikobehaftetheit zu reflektieren, um daraus ihre Bedeutung für gesellschaftliche Teilhabefragen ableiten zu können (vgl. Beranek 2020, S. 427).

Auch die digitale Präsenz und Sichtbarkeit von Einrichtungen nach Außen gewinnt – neben der Relevanz für ihre Zielgruppe – durch die fortschreitende ökonomische Betrachtung der Sozialen Arbeit an Stellenwert. Auch dadurch werden neue Anforderungen an Pädagog_innen gestellt (vgl. ebd., S. 431). So müssen sich Fachkräfte neben ihrer pädagogischen Arbeit mit technischen Kompetenzen, Datenschutz sowie Vor- und Nachteilen von Social Media auseinandersetzen (vgl. Kutscher et al. 2020, S. 9). Darüber hinaus fehlen den sozialen Einrichtungen häufig die finanziellen Ressourcen für den zusätzlichen Zeitaufwand oder überhaupt für die technische Ausstattung (vgl. Iske & Kutscher 2020, S. 124).

Grundsätzlich kann festgestellt werden, dass es bislang nur wenige Studien zur Digitalisierung in der Sozialen Arbeit gibt (Evans und Hilbert 2020, S. 78). Im Hinblick auf das Arbeitsfeld der Sozialen Arbeit stellten Klein (2008) sowie Kutscher und Zimmermann (2011)^1^ heraus, dass Angebote der Onlineberatung von sozial benachteiligten Adressat_innen wenig in Anspruch genommen werden. Dass die Digitalisierung auch Chancen für benachteiligte Menschen bietet, zeigt die Studie von Friedrichs-Liesenkötter, Lemke und Hüttmann (2022) zu geflüchteten Jugendlichen. Unter anderem konnten sprachliche Barrieren durch die digitale Unterstützung abgebaut werden. Entscheidend für das Entgegenwirken der Reproduktion ungleicher Teilhabechancen scheint eine pädagogische Betreuung zu sein. Diese Annahme bestätigen auch Studien über Lockdowns der Covid-19-Pandemie im schulischen Kontext (vgl. Wößmann et al. 2020; Eickelmann und Gerick 2020).

Im Folgenden sollen nun Perspektiven von Fachkräften, die sich in der außerschulischen kulturellen Bildungsarbeit verorten und in die pädagogische Arbeit mit Jugendlichen eingebunden sind, auf die Digitalisierung ihrer Angebote in den Blick genommen werden. Im Fokus steht dabei die Frage, wie sie sich selbst zur Integration digitaler Angebote in ihrer pädagogischen Praxis positionieren und wie sich dies auf ihr professionelles Selbstverständnis auswirkt.

## Das Forschungsprojekt

Das vom Bundesministerium für Bildung und Forschung geförderte Verbundprojekt „Kulturell-musische Bildung Jugendlicher des ländlichen Raums“ (Kumulus) der Martin-Luther-Universität Halle-Wittenberg und dem Deutschen Jugendinstitut untersucht die Bedingungen und Praktiken kulturell-musischer Bildung für und von Jugendlichen im ländlichen Raum (vgl. Grunert et al. 2021). Hierfür wurden unter anderem 23 Expert_innen-Interviews von Oktober 2020 bis Ende 2021 geführt sowie weitere 22 Leitfadeninterviews mit Jugendlichen im Erhebungszeitraum von November 2021 bis August 2022. Die Untersuchungen fanden in zwei ländlichen Regionen in Ostdeutschland statt, die nach der Kategorisierung des Bundesinstituts für Bau‑, Stadt- und Raumforschung (2019) als sehr peripher gelten. Ausgewertet haben wir die Interviews nach der Grounded Theory (vgl. Corbin & Strauss 2015). Fokus dieses Beitrages bilden vier Interviews mit Expert_innen. Die Fälle kontrastieren deutlich Umgangsweisen mit den Digitalisierungsanforderungen, mit denen die Expert_innen in ihrer professionellen Praxis vor dem Hintergrund der Schließung von Räumen im Zuge der Corona-Pandemie-Maßnahmen konfrontiert wurden. Vor Beginn der Pandemie haben hier digitale Technologien zur Unterstützung der pädagogischen Arbeit kaum eine Rolle gespielt.

## Aufrechterhaltung grundlegender gesellschaftlicher Teilhabe im Jugendtreff

Die Grenzen der Herstellung und Aufrechterhaltung pädagogischer Arbeitsbündnisse über digitale Raumformate zeigt sich vor allem dann, wenn die Arbeit mit sozial benachteiligten Kindern und Jugendlichen den Kern des professionellen Handelns darstellt. So verorten sich die beiden Kinder- und Jugendtreffs – die nun im Mittelpunkt stehen – selbst in einem „sozialen Brennpunkt“. (Vogler, Z. 70, 330; Hejduska, Z. 310 im Transkript des geführten Interviews). Aufgrund der fehlenden öffentlichen Mobilitätsinfrastruktur in den ländlichen Regionen erreichen sie ausschließlich Kinder und Jugendliche aus dem jeweiligen Wohngebiet. Insbesondere die fehlende technische Ausstattung der Kinder und Jugendlichen schränkt die pädagogische Interaktions- sowie Handlungsfähigkeit nach der Schließung der Jugendtreff-Räume deutlich ein. Infolgedessen stellte sich bei beiden Jugendtreffs nicht die Frage, inwiefern sie digitale Angebote gestalten, sondern wie sie pädagogische Arbeitsbündnisse überhaupt aufrechterhalten und weiterhin Zugänge zu den Jugendlichen finden können. So sieht Karl Vogler die Fortsetzung seiner pädagogischen Arbeit bei geschlossenem lokal verortetem Raum in Outdoor-Angeboten. Hierbei greift er auf klassische Outdoor-Angebote zurück, wie Riesenmikado, diverse Bastelangebote, interaktive Spiele etc. Diese wurden allerdings nur von einem Viertel der Kinder genutzt, während die Jugendlichen gar nicht kamen. Begründungsfigur für das Vorgehen ist sein professionelles Selbstverständnis, in dem der Ort des Jugendclubs als Ermöglichungsraum für soziale Interaktionen unter körperlich Anwesenden eine zentrale Rolle spielt. Dabei geht es K. Vogler „in erster Linie darum, dass sie überhaupt einen Treffpunkt haben, wo sie sich auch mit anderen Kindern und Jugendlichen ja treffen können ähm auch wieder unter den besonderen Bedingungen von Corona dass sie dort eben die Möglichkeit haben der ähm ja sozialen Isolation ähm son zu entgehen.“ (Vogler, Z. 41–45 im Transkript des geführten Interviews).

Deutlich wird, dass digitale Formate bislang keine Rolle in der Ausgestaltung des pädagogischen Handelns gespielt haben und er dies auch in seinem professionellen Selbstverständnis ausklammert. Für ihn gehören vor allem die Betreuung der PC-Benutzung, Kochabende, Unterstützung bei den Hausaufgaben, an Feiertagen angelehnte Veranstaltungen und dazugehörigem Basteln „zur pädagogischen Arbeit, die uns ausmacht“. (Vogler, Z. 65–66 im Transkript des geführten Interviews). Legitimiert wird dies durch die dankbare Annahme der Angebote von den Adressat_innen. Entsprechend verankerte sich seine Strategie während der Covid-19-Pandemie in der Hoffnung auf niedrige Inzidenzen, damit der Jugendclub weiterhin geöffnet bleibt und „alle Kinder und Jugendliche [an] alle[n] Angebote[n] teilnehmen“ können. (Vogler, Z. 151 im Transkript des geführten Interviews).

Im Jugendtreff von Thomas Hejduska zeigen sich ebenso ungleiche Zugangs- und Teilhabechancen der Jugendlichen. So hatten einige Jugendliche Schwierigkeiten, überhaupt am digitalen Schulunterricht teilnehmen zu können. Aufgrund des fehlenden ökonomischen Kapitals der Familien hatten die Jugendlichen teilweise einen Internetzugang nur über die Handys der Eltern. Dadurch standen die Jugendlichen für den Pädagogen nicht als Adressat_innen des Jugendtreffs im Fokus, sondern in ihrer Rolle als Schüler_innen. Durch sein hohes persönliches Engagement und die Nutzung des eigenen Datenvolumens über sein privates Handy ermöglichte er den Jugendlichen die Teilnahme am Schulunterricht.

Dennoch ist bei beiden Fachkräften die Pflege von Social Media-Kanälen in ihr Selbstverständnis pädagogischer Arbeit eingebettet, auch wenn sie hierbei unterschiedliche Schwerpunkte setzen. Im Jugendtreff von K. Vogler werden die Sozialen Medien vor allem für die Außendarstellung der Angebote genutzt. Zudem wurde diese Arbeit an Ehrenamtliche ausgelagert. T. Hejduska hingegen betreibt über die Sozialen Medien Beziehungsarbeit mit den Jugendlichen. Dabei beugt er gleichzeitig einer Entgrenzung seiner professionellen Tätigkeit vor, indem er den Jugendlichen transparent macht, zu welchen Zeiten er über sein Diensthandy bei Instagram verfügbar ist. Diese Präsenz auf Social-Media-Kanälen ist allerdings auch mit der Öffnung der Jugendtreff-Räume wieder zurückgegangen.

Hinsichtlich der Nutzung von digitalen Möglichkeitsräumen nimmt Hejduska eine ambivalente Haltung ein. Er sieht zwar die Notwendigkeit der digitalen Weiterentwicklung des Jugendtreffs aufgrund gesellschaftlicher Wandlungsprozesse. Allerdings hat er ein klares Ziel nach der Pandemie: „[N]ach der Pandemie ist das muss das sofort der analoge Ausweg sein weil die sind auch irgendwann sind sie halt auch einfach tatsächlich übersättigt.“ (Hejduska, Z. 416–417 im Transkript des geführten Interviews).

Seine ablehnende Haltung begründet Hejduska auch mit seinen fehlenden Kompetenzen bzgl. digitaler Formate. So muss sich der Sozialpädagoge aufwändig in die Social Media-Plattformen einarbeiten, die die Jugendlichen alltäglich und selbstverständlich verwenden. Das weist auch auf die fehlenden Ressourcen zur Auslagerung dieser Tätigkeit an eine andere Fachkraft oder Ehrenamtliche hin, wie es in dem Jugendtreff von K. Vogler der Fall ist. So stellt es für T. Hejduska eine Lösung dar, sein Wissen auf eine Plattform zu beschränken. Dieses nutzt er vordergründig zur Kontrolle, indem er nun über die Möglichkeit des potenziellen Eingreifens in Hinblick auf die gesehenen und geteilten Beiträge der Jugendlichen verfügt.

## Herstellung von digitalen Teilhaberäumen im Jugendtreff

Ein Gegenbeispiel zu den vorherigen Fällen stellt ein Jugendtreff dar, der verschiedene soziale Milieus erreicht: „[D]ie denn aber auch sagen ‚ey wir hängen mit den Kids ab die sozial schwach sind und wo mans auch sieht beim Äußerlichen und sind trotzdem zu denen nett‘ ja also von daher ich sag mal bei uns läuft das hier wirklich mit als großes Miteinander hier ist wirklich jeder am am Tisch willkommen kann sich mit dransetzen und profitiert dann von den anderen.“ (Stein, Z. 404–408 im Transkript des geführten Interviews).

Lukas Stein etabliert während der Covid-19-Pandemie diverse digitale Angebote, wodurch er einen Zugewinn von Jugendlichen verzeichnen kann. Dabei wählt er einen lebensweltorientierten und kreativen Zugang, bspw. Livestreams, online „zocken“ (Stein, Z. 90 im Transkript des geführten Interviews), Zuschalten von Jugendlichen beim Hausaufgaben machen etc. Die Öffnung von digitalen Formaten erscheint als reibungsloser Prozess und weniger spannungsgeladen als bei dem Beispiel Hartmann. Dadurch konnte L. Stein, wie er selbst sagt, seine Angebotszahl um 70 % steigern. An dieser Stelle sei zu erwähnen, dass er vom Alter her den Jugendlichen wesentlich nähersteht als Hejduska und mit den digitalen Möglichkeiten mehr aufgewachsen ist. Dennoch adressiert er die Jugendlichen als Expert_innen für digitale Formate, holt sich bspw. beim Posten auf Instagram ihre Meinung ein und setzt die Ratschläge der Jugendlichen um, ohne dass dies im Widerspruch zu seinem professionellen Selbstverständnis steht: „[W]enn wir jetzt zum Beispiel Livestream machen über das und das Thema neulich zum Beispiel wir haben ja ne Clique von Fünfzehn Sechzehnjährigen die sind voll im Thema drin schlagen wir denen das vor fragen dann auch mal so nach der Meinung ‚was meint ihr zieht das‘ ‚ja nee ist altbacken macht mal so und so das kommt jetzt an das müsst ihr jetzt mal auschecken‘ dann versuchen wir das umzusetzen funktioniert.“ (Stein, Z. 372–377 im Transkript des geführten Interviews).

Darüber hinaus hat L. Stein wie T. Hejduska die Beziehungspflege während der Pandemie in den digitalen Raum verlagert und kommuniziert mit den Jugendlichen über Messenger. Das pädagogische Arbeitsbündnis wird damit gerade über den temporären Verlust der Kommunikation unter körperlich Anwesenden neu hergestellt: als Spannungsverhältnis von pädagogisch initiierter, stellvertretender Bereitstellung jugendbezogener Räume sowie der anvisierten partizipativen Gestaltung und Aneignung durch die Jugendlichen selbst. Darüber hinaus dient der Social-Media-Kanal dazu, aktuelle Informationen über den Jugendclub nach außen zu tragen und wird über die Pandemie hinaus gepflegt.

Hervorzuheben ist, dass L. Stein nicht nur die bisherigen Praktiken auf digitale Formate überträgt, sondern die Kernbestandteile seiner pädagogischen Tätigkeit neu denkt. Dies integriert er in sein professionelles Selbstverständnis, dass auf die Ermöglichung heterogener und partizipativer Prozesse der Raumaneignung durch die Jugendlichen gerichtet ist. Digitale Formate stellen hier nicht nur eine Notlösung dar, sondern es zeichnet sich ab, dass sie auch weiterhin zu einem integrativen Teil seines pädagogischen Handelns gehören werden.

## Herstellung von Lernräumen in der Musikschule

Felix Hartmann leitet eine Musikschule und ist persönlich in Unterrichtsprozesse involviert. Als Reaktion auf die Schließung der Musikschule durch den Lockdown der Covid-19-Pandemie startet er nach kurzer Wartezeit den Musikschulunterricht im Online-Format. Dies erfolgt aus der Notsituation heraus, um als Musikschule handlungsfähig und identifizierbar zu bleiben. Im Hinblick auf das Engagement der Fachkräfte an der Musikschule, den Unterricht im digitalen Format anzubieten, zeigen sich große Unterschiede. So entstehen auf der organisationalen Ebene bürokratische und infrastrukturelle Hürden bei der Verlagerung des Musikunterrichts – der sich durch körperliche Anwesenheit auszeichnet – in einen über digitale Medien hergestellten virtuellen Raum. Einerseits schildert der Musikschulleiter einen unkomplizierten Übergang, andererseits wird deutlich, dass der Erfolg des digitalen Musikunterrichts abhängig vom individuellen Engagement der Lehrenden ist. Dabei wird er auch mit der Abwehr von Lehrenden konfrontiert.

Deutlich wird in seiner Erzählung, dass die Umstellung auf ein digitales Format eine Neuaushandlung der Arbeitsbündnisse zwischen Schüler_innen und Lehrer_innen sowie eine Neubestimmung des professionellen Selbstverständnisses notwendig macht. Insbesondere gründet sich dies auf die Wahrnehmung einer Differenz beim Verfügen über Raumwissen im Digitalen zwischen Schüler_innen und Lehrenden. So ließ sich die pädagogische Situation zum Teil nur darüber aufrechterhalten, dass Musikschüler_innen bei der technischen Umsetzung der Videokonferenz Unterstützung leisteten: „Ich hab gestaunt auch bei vieln Kindern und Jugendlichen die warn da sehr schnell dazu in der Lage auch welche aus der fünften Klasse und so die ham mir dann auch gesagt Mensch Herr Hartmann Sie können ja dort nochmal draufdrücken und dann könn wir das so und so machn also es geht schon das funktioniert schon“. (Hartmann, Z. 350–354 im Transkript des geführten Interviews).

F. Hartmann reflektiert sozial ungleiche Zugangsmöglichkeiten zum Musikunterricht und sucht hierfür auch nach Lösungen. Im Hinblick auf den digitalen Musikunterricht sieht er es allerdings als Selbstverständlichkeit an, dass die Anschaffung eines technischen Geräts, das die Teilhabe am digitalen Unterricht ermöglicht, in der Verantwortung der Familie liegt: „[U]nd diese Gerätschaften ja gut die musste dann jeder ja zu Hause irgendwie natürlich vorhalten is ja klar.“ (Hartmann, Z. 345–347 im Transkript des geführten Interviews). Entsprechend sieht er es nicht in seinem Verantwortungsbereich, für Zugänge auf Seiten der Schüler_innen zu sorgen und diese Benachteiligung zu bearbeiten. Dementsprechend werden die Jugendlichen und ihre Familien dafür responsibilisiert und ungleiche Zugangschancen eher ausgeblendet.

In der Erzählung fokussiert F. Hartmann hingegen die Jugendlichen, bei denen der Umstieg auf den digitalen Unterricht problemlos funktioniert hat. Kontrastiert werden lediglich die Qualitätsunterschiede der technischen Geräte, wobei er die engagierten Familien mit höherem ökonomischem Kapital positiv hervorhebt. Eine kritische Auseinandersetzung mit dem Phänomen der digitalen Ungleichheit, wie sie Iske und Kutscher (2020) fordern, findet eher nicht statt: „[J]a also muss ich sagn und es gab auch – eh gibt natürlich große Tonqualitätsunterschiede was aber nicht an der Internetverbindung lag sondern mehr an den Geräten an den Endgeräten ja ist natürlich ein Unterschied wenn ich jetzt so *n* relativ eh einfaches Handy habe oder ich hab dann eben doch manche Kinder oder Jugendlichen hatten sich dann extra noch zu ihrem Laptop noch ein Mikrofon besorgt ein richtiges wie so ein Studiomikrofon und eh sogar noch manchmal ein bissel ausgeleuchtet so und so @haben sich manche@ richtig eh richtig Mühe gegeben ja das war dann richtig gut.“ (Hartmann, Z. 365–372 im Transkript des geführten Interviews).

Je nach ökonomischen Voraussetzungen kann somit ein qualitativ unterschiedlicher Musikunterricht gewährleistet werden. Zudem sind Zugänge im ländlichen Raum – in dem die Musikschule agiert – immer auch mit regionalen Ungleichheiten verbunden, so dass auch der fehlende Breitbandausbau zur Exklusion aus dem pädagogischen Setting führen kann. F. Hartmanns Haltung gegenüber einer nachhaltigen Öffnung der Musikschule in digitale Formate lässt sich als ambivalent einschätzen. Einerseits verweist er aufgrund der bisherigen Erfahrungen darauf, bei erneuten Schließungen unkompliziert auf das Onlineformat zurückgreifen zu können, bspw. bei Quarantänefällen. Dadurch betrachtet er aber dieses Angebot als Ausnahme von der Normalität und markiert die Musikschule in ihrer lokalen Verankerung als zentralen Ort. Andererseits „scheinen die positiven Erfahrungen mit der Praktikabilität digitalen Musikunterrichts sowie die Wahrnehmung der wachsenden gesellschaftlichen Bedeutung digitaler Räume und des damit verbundenen Anpassungsdrucks eine dauerhaft veränderte Raumkonstellation nahezulegen“. (vgl. Grunert et al. 2022, S. 97) Hierbei kann der Ort der Musikschule zwar weiterhin das Zentrum bilden, wird aber punktuell für digitale Technologien geöffnet. Darin gerät er vor allem von Seiten der Eltern unter Druck, die den Rückgriff auf digitale Formate auch über die Pandemie hinaus einfordern. Dadurch möchten sie lange Fahrtwege, die in der Region für die Ermöglichung des Musikunterrichts notwendig sind, punktuell vermeiden.

## Fazit

In den Fallbeispielen zeigen sich die bislang weitgehend nicht anzutreffende respektive nicht realisierte Digitalisierungsprozesse in der Sozialen Arbeit und Kulturellen Bildung. Deutlich werden zudem die Möglichkeiten und Grenzen der pädagogischen Fachkräfte mit den Digitalisierungsanforderungen im Zuge der Covid-19-Pandemie. Der geforderte lebensweltorientierte Zugang im Hinblick auf digitale Angebote (vgl. Tillmann 2020; Iske & Kutscher 2020) zeigt vor allem der Fall L. Stein. In der Konsequenz macht sich das positiv durch die steigenden Besucher_innenzahl des Jugendclubs bemerkbar. Er meint, die digitalen Angebote an die Lebensrealität der Zielgruppe anpassen zu müssen, damit diese auch angenommen werden. Hierbei rückversichert er sich bei den Jugendlichen, ob die Gestaltung seiner digitalen Formate sie in deren Lebenswelt abholen. Er bleibt somit im persönlichen Kontakt mit den Jugendlichen und kann pädagogische Arbeitsbündnisse aufrechterhalten und sogar weiter ausbauen. Ferner scheint der temporäre Rollentausch – die Adressat_innen als Expert_innen zu adressieren – Teil seines professionellen Selbstverständnisses zu sein.

Die drei weiteren Fälle bestätigen hingegen die These, dass sich ohnehin vorhandene soziale Ungleichheiten über die Notwendigkeit, auf digitale Formate zurückgreifen zu müssen, noch weiter verstärken. Insbesondere bei der Musikschule macht sich die fehlende Reflektion der ungleichen sozialen Teilhabe an digitalen Angeboten bemerkbar. So ermöglicht die Musikschule den Jugendlichen – die über die technischen Möglichkeiten verfügen – zwar die Zugänge zu den Lernräumen, aber nicht grundsätzlich die Teilhabe. Es geht eher darum, musikalische Angebote den digitalen Möglichkeiten der Musikschule und nicht die digitalen Angebote den Möglichkeiten der Jugendlichen entsprechend zu gestalten. Die Verantwortung für die Organisation der notwendigen technischen Ausstattung wird den Adressat_innen überlassen, was zum Ausschluss der Jugendlichen führt, deren Familien dies nicht erfüllen können. Gleichzeitig stehen regionale Ungleichheiten im Sinne unzureichender Netzzugänge den Teilhabemöglichkeiten entgegen.

Insbesondere die Sozialpädagog_innen in den Jugendtreffs verfügen über ein Wissen bezüglich der Lebenswelt ihrer Zielgruppe und daraus resultierende soziale Ungleichheiten. Entsprechend versuchen sie, ihre Angebote den jeweiligen Bedingungen anzupassen. Hierbei scheinen die pädagogischen Fachkräfte digitale Angebote kaum in den Blick zu nehmen – sei es aufgrund der fehlenden technischen Ausstattung der Jugendlichen, unzureichender Kompetenzen zum Umgang mit digitalen Medien oder der fehlenden Integration dieser in ihr professionelles Selbstverständnis. Durch die fehlende Erreichbarkeit der Jugendlichen zu Zeiten der geschlossenen Räume lässt sich allerdings fragen, ob niedrigschwellige digitalisierte Angebote Jugendliche in ihrer Lebenswelt mehr abgeholt und an den Jugendclub gebunden hätten, wo ihnen zumindest digital ein sozialer Austausch möglich gewesen wäre.

Die Fallbeispiele zeigen, dass sich das Anforderungsprofil der Fachkräfte erweitert, sobald digitale Angebote in die soziale – pädagogische – Praxis eingebunden werden. Dafür benötigt das pädagogische Personal sowohl Qualifizierungsmöglichkeiten im Bereich der Medienkompetenz als auch zeitliche und finanzielle Ressourcen. Die Auseinandersetzung mit Digitalisierungsfragen war den interviewten Fachkräften vor allem auch aufgrund der geschlossenen Räume und des fehlenden pädagogischen Alltags möglich. Allerdings wird auch sichtbar, dass digitale Angebote vom Engagement Einzelner – wie bei L. Stein – abhängig sind und ein geringer Personalschlüssel oder ein größerer Generationsunterschied – wie bei K. Vogler und T. Hejduska – entscheidend sein können. Dies untermauert die Forderung (vgl. Iske & Kutscher 2020), dass insbesondere auf der organisationalen Ebene Bedingungen für Fachkräfte zu schaffen sind, Digialisierungsprozesse zu realisieren.

## Footnotes

1 Kutscher, N., & Zimmermann, K. (2011). Ergebnisbericht zur UserInnenbefragung der kidshotline. Nutzungsweisen, Präferenzen und Beteiligungswünsche älterer und jüngerer NutzerInnen im Vergleich. Unveröffentlichtes Manuskript.
